# Association of Sociodemographic Variables and Healthy Habits with Body and Visceral Fat Values in Spanish Workers

**DOI:** 10.3390/medicina61010150

**Published:** 2025-01-17

**Authors:** María Gordito Soler, Ángel Arturo López-González, Pedro Juan Tárraga López, Emilio Martínez-Almoyna Rifá, Cristina Martorell Sánchez, María Teófila Vicente-Herrero, Hernan Paublini, José Ignacio Ramírez-Manent

**Affiliations:** 1 Pharmaceutical, 41013 Seville, Spain; mgorditosoler@hotmail.com; 2 Investigation Group ADEMA SALUD, University Institute for Research in Health Sciences (IUNICS), 07120 Palma, Spain; emilio@udemax.com (E.M.-A.R.); c.martorell@eua.edu.es (C.M.S.); correoteo@gmail.com (M.T.V.-H.); h.paublini@eue.edu.es (H.P.); joseignacio.ramirez@ibsalut.es (J.I.R.-M.); 3 Faculty of Dentistry, University School ADEMA, 07009 Palma, Spain; 4 Faculty of Medicine, University of the Castilla la Mancha, 02071 Albacete, Spain; pjtarraga@sescam.jccm.es; 5 Balearic Islands Health Service, 07003 Palma, Spain; 6 Faculty of Medicine, University of the Balearic Islands, 07122 Palma, Spain

**Keywords:** body fat, visceral fat, sociodemographic variables, smoking, physical activity

## Abstract

*Background and Objectives*: The accumulation of fat in the body, especially visceral fat, is associated with various cardiometabolic conditions such as diabetes mellitus and fatty liver. The reasons for the accumulation of this fat are diverse. Some studies, also in the working population, have shown a clear association between sociodemographic variables and health habits with scales that assess overweight and obesity. This study aims to determine how certain sociodemographic variables, such as age, gender, and socioeconomic level, as well as certain healthy habits like physical activity and tobacco consumption, affect the levels of body and visceral fat. *Materials and Methods*: We conducted a descriptive and cross-sectional study involving 8590 Spanish workers. The percentage of body and visceral fat was measured using a bioimpedance analysis with a Tanita DC 430MA device. *Results*: Both the average values and the prevalence of elevated body and visceral fat increase with age and decrease with social class and lower levels of physical activity. These values are higher in smokers. A multivariate analysis shows that the variables most influential in increasing the risk of high levels of both body and visceral fat are age and low levels of physical activity. *Conclusions*: The profile of a person at high risk of having elevated body and visceral fat levels is an older male with a low socioeconomic status who smokes and leads a sedentary lifestyle.

## 1. Introduction

The distribution and accumulation of body fat, particularly visceral fat, is a topic of significant interest due to its strong association with various metabolic and cardiovascular diseases. Visceral fat, primarily located around internal organs, has been identified as a key risk factor for the development of metabolic syndrome, type 2 diabetes, and cardiovascular diseases [1]. Although fat accumulation is influenced by a combination of genetic and environmental factors, recent studies have highlighted the importance of sociodemographic variables and lifestyle habits in determining the levels of body and visceral fat [2,3].

Age is one of the most relevant factors in the accumulation of body and visceral fat, a process influenced by various physiological and metabolic changes associated with aging, which causes a shift in fat distribution, with a proportional increase in visceral fat compared to subcutaneous fat [4]. As people age, they experience a decrease in muscle mass, known as sarcopenia, which reduces energy expenditure and facilitates fat accumulation, particularly in the abdominal region. This is partly due to the decline in physical activity, which is often observed in older adults, contributing to weight gain and altered fat distribution. Additionally, metabolic changes, such as a decrease in basal metabolic rate, also favor fat accumulation [5]. Visceral fat, which accumulates around internal organs, is closely associated with a higher risk of metabolic and cardiovascular diseases. Furthermore, hormonal changes, such as the decrease in growth hormone levels and estrogen in women, and testosterone in men, favor abdominal fat storage [6].

Sex is another crucial determinant in the distribution of body fat. Women tend to accumulate more subcutaneous fat, particularly in the gluteofemoral region, while men are more prone to accumulating visceral fat [7]. This difference is largely mediated by hormonal levels, particularly estrogen in women and testosterone in men [8]. However, postmenopausal women experience a shift toward greater visceral fat accumulation, making them more susceptible to diseases associated with central obesity [9].

Socioeconomic status, often reflected by social class, also influences body and visceral fat levels. Individuals from lower socioeconomic backgrounds tend to have higher prevalence rates of obesity and visceral fat accumulation, which has been attributed to factors such as lower health education, reduced access to healthy foods, and a higher prevalence of risk behaviors like sedentary lifestyles [10]. Epidemiological studies have shown that individuals from lower social strata are at significantly higher risk of developing obesity-related diseases, including cardiovascular diseases and type 2 diabetes [11].

Tobacco consumption has paradoxical effects on body fat. While smokers tend to have a lower body mass index (BMI) compared to non-smokers, their fat distribution is more unfavorable, with a higher proportion of visceral fat [12]. It has been suggested that nicotine has a catabolic effect on fat mass but, at the same time, promotes the redistribution of fat toward the abdominal region [13]. Furthermore, smoking cessation is often accompanied by weight gain, which could lead to greater visceral fat accumulation if not accompanied by increased physical activity or dietary changes [14].

Physical activity is one of the most important modifiable factors in the regulation of body and visceral fat. Regular physical activity, especially aerobic and resistance exercises, has been associated with a significant reduction in visceral fat, even without substantial weight loss [15]. This is because exercise increases insulin sensitivity and improves fat metabolism, favoring the use of visceral fat as an energy source [16]. Additionally, physical activity also has a preventive effect by reducing fat accumulation with age and mitigating the negative effects of other risk factors such as sedentary behavior and unhealthy dietary patterns [17].

The aim of the study was to explore the association between sociodemographic variables (age, sex, and socioeconomic status) and health habits (smoking and physical activity) with body and visceral fat values determined by bioimpedance.

## 2. Methods

### 2.1. Participants

**Figure 1:**
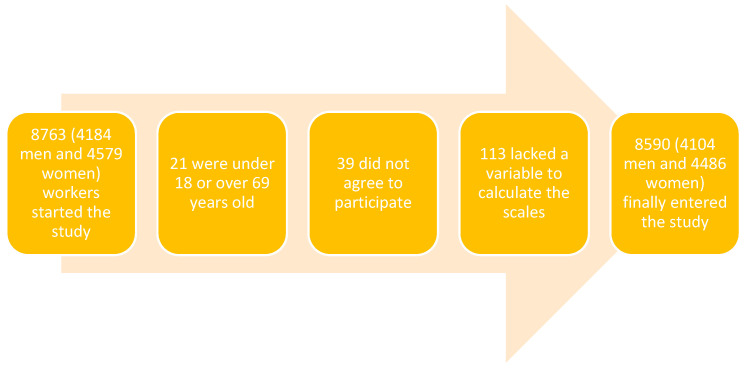
Flowchart.

A cross-sectional and descriptive study involving 8590 Spanish workers in the Balearic Islands was conducted. The study sample consisted of all workers who underwent occupational health examinations between January 2019 and December 2020. The age of the workers was 18 to 69 years, as this is the legal age of public workers in our country. Please refer to the flow chart in Figure 1 for further details.

Inclusion Criteria:

- Aged between 18 and 69 years.
- Willing to participate in the research.
- Consented to their data being used for epidemiological research.
- Employment with one of the companies participating in the study and not being on temporary disability leave at the time of the study.

Exclusion Criteria:

- Aged under 18 years or over 69 years.
- No employment contract with a participating company.
- Did not provide informed consent to participate in the study.
- Did not authorize the use of their data for epidemiological purposes.

### 2.2. Determination of Variables

All measurements, whether anthropometric (height, weight, and waist circumference), analytical, or clinical, were conducted by occupational health professionals from the participating companies following process standardization to prevent interobserver bias:

These included age, sex, socioeconomic status, regular physical exercise, days of physical exercise per week (physical activity was determined using the validated International Physical Activity Questionnaire (IPAQ), establishing three categories) [18], and smoking status.

Socioeconomic class was determined using the recommendations of the Spanish Society of Epidemiology, based on the 2011 National Classification of Occupations. Class I includes managers, directors, and university professionals (upper class); class II consists of intermediate vocations and self-employed individuals (middle class); and class III comprises manual workers (lower class) [19].

#### 2.2.1. Anthropometric Determinations

These included measurements of weight, height, waist and hip circumference, and both systolic and diastolic blood pressure.

Height (in cm) and weight (in kg) were measured using an SECA 700 scale, adhering to the International Society for the Advancement of Kinanthropometry (ISAK) standards for anthropometric assessment [20]. Waist circumference was measured with the subject standing, feet together, and abdomen relaxed, using a tape measure parallel to the floor at the midpoint between the last palpable rib and the iliac crest [21]. Body and visceral fat percentages were determined using a bioimpedance analysis with a Tanita DC 430MA model. High values of body and visceral fat are considered those indicated by the bioimpedance scale (from 10 for visceral fat, with body fat values varying according to age).

#### 2.2.2. Clinical Determinations

Blood pressure was measured after the subject had rested for 10 min, seated with uncrossed legs, using an OMRON-M3 model blood pressure monitor. Three measurements were taken at one-minute intervals, and the average of the three readings was calculated.

#### 2.2.3. Analytical Determinations

These included fasting blood glucose, lipid profile, and hepatic enzymes.

Blood samples were collected after a minimum of 12 h of fasting and processed within 48 to 72 h. The levels of triglycerides, total cholesterol, and blood glucose were measured using automated enzymatic procedures. The dextran sulfate-MgCl_2_ precipitation technique was used to measure HDL-cholesterol. LDL-cholesterol was calculated indirectly using the Friedewald formula, which is reliable only when triglyceride levels do not exceed 400 mg/dL. The unit of measurement for all analytical parameters is mg/dL [22]:

LDL = Total cholesterol − HDL-c + Triglycerides/5

Anyone who had smoked at least one cigarette in the past month (or its equivalent in other forms of consumption) or had quit smoking less than a year prior was considered a smoker.

### 2.3. Statistical Analysis

A descriptive analysis of categorical variables was performed using frequencies and distributions. The Kolmogorov–Smirnov test assessed the normality of quantitative variables, followed by the calculation of means and standard deviations. For the bivariate analysis, Student’s *t*-test was used to compare means, while the chi-square test assessed proportions. Variables associated with body and visceral fat values were analyzed using a binary logistic regression model, with model fit evaluated using the Hosmer–Lemeshow test. A stratified analysis identified potential confounding factors, but no variables showed significant confounding effects. The statistical analysis was performed using SPSS version 29.0, with a significance level set at *p* < 0.05.

## 3. Results

The anthropometric and clinical details of the study participants are presented in Table 1. The analysis included a total of 8590 workers (4104 men, 47.8%, and 4486 women, 52.2%). The average age of the participants was slightly above 41, with most of the participants falling within the 30–49 age range. The majority of the workers belonged to social class I. In both men and women, just over 15% were smokers. Among the men and women, 25.9% and 35.1%, respectively, did not engage in regular exercise.

The average values of both body fat and visceral fat, in both sexes, increase in parallel with age. An increase in these variables is also observed as socioeconomic status decreases, with the highest values seen in individuals from socioeconomic class III. The amount of physical exercise also has an impact, with greater levels of physical activity associated with lower body and visceral fat values. Tobacco consumption increases both parameters. Body fat values are higher in women, whereas visceral fat values are higher in men. In all cases, the differences are statistically significant (*p* < 0.001) (Table 2).

When we assess the prevalence of very high body fat values and high visceral fat, we can observe a trend similar to that already mentioned for average values; that is, an increase in prevalence with aging, as we go down the social and economic levels, among smokers, and in people with a sedentary lifestyle. The prevalence of high values for both parameters is higher in men. Similarly, the differences observed show a high level of significance (*p* < 0.001). (Table 3).

In the analysis using multinomial logistic regression (Table 4), we observe that all variables, both sociodemographic and healthy habits, increase the risk of having very high levels of body fat and high levels of visceral fat. Among these variables, age and physical activity show the greatest influences. In all cases, the level of statistical significance is very high (*p* < 0.001).

## 4. Discussion

The accumulation of body fat, particularly visceral fat, is closely associated with an increased risk of cardiometabolic diseases, including type 2 diabetes, cardiovascular diseases, and non-alcoholic fatty liver disease. A variety of sociodemographic and behavioral factors influence the distribution and accumulation of fat in the body. This study examines how age, sex, social class, smoking, and physical activity affect the levels of body and visceral fat. The results underscore the importance of considering these factors in order to effectively address health risks related to obesity. The averages and prevalence of elevated body and visceral fat values are influenced by all the variables analyzed, including sociodemographic factors (age, sex, and social class) and health behaviors (smoking and physical activity).

Numerous studies have established age as a key determinant in the accumulation of body and visceral fat. As individuals age, there is a marked increase in total body fat, particularly in visceral fat. This phenomenon can be attributed to hormonal changes, a decline in basal metabolic rate, and a reduction in physical activity levels as people grow older [4,23]. The present study found a significant correlation between advancing age and elevated levels of both body and visceral fat, which aligns with the existing literature. The redistribution of fat towards visceral areas with age is particularly concerning, as visceral fat is more closely associated with metabolic risks [24].

Sex also plays a critical role in fat distribution patterns. It has been well documented that women tend to accumulate more subcutaneous fat, whereas men generally exhibit higher levels of visceral fat [25]. This distribution pattern may be influenced by hormonal differences, such as the effects of estrogens and androgens on fat distribution [26]. In our study, men exhibited significantly higher levels of visceral fat compared to women, a finding consistent with previous research [27]. These sex differences underscore the need for tailored approaches in obesity prevention and treatment strategies for men and women.

Social class is another important factor influencing fat accumulation patterns. Individuals from lower socioeconomic backgrounds are more likely to experience higher rates of obesity, partly due to limited access to healthy foods, lower levels of education, and fewer opportunities for physical activity [28,29]. In the current study, individuals in lower social classes exhibited higher body and visceral fat values, aligning with previous research linking poverty to a higher risk of obesity and related diseases [30]. Public health policies should focus on improving access to healthy food and physical activity opportunities for low-income populations.

Historically, tobacco consumption has been viewed as a factor contributing to weight reduction; however, recent studies suggest that smoking may be associated with increased visceral fat accumulation [31]. Potential mechanisms include alterations in fat metabolism and the development of insulin resistance induced by smoking [32]. In this study, smokers exhibited significantly higher levels of visceral fat, supporting the hypothesis that smoking contributes to an adverse metabolic profile [33]. These findings emphasize the need to incorporate smoking cessation as a central component of obesity management strategies.

Regular physical activity is a critical factor in weight management and the reduction in both body and visceral fat. Evidence suggests that aerobic and resistance exercise not only helps reduce subcutaneous fat but also decreases visceral fat, which is essential for mitigating cardiometabolic risks [15,34]. Our study found that lower levels of physical activity were associated with higher body and visceral fat values, which is consistent with numerous studies advocating for the promotion of physical activity to prevent obesity [35]. Implementing structured exercise programs in community and workplace settings may be an effective strategy to combat obesity and improve public health.

In our study, the large sample highlights the importance of considering lifestyle habits and educational level as key factors in the prevention of obesity-related risks. Significant findings between sexes underscore the need for personalized interventions for men and women, optimizing strategies according to their needs. Individuals from lower social classes showed higher levels of body and visceral fat, in line with research associating poverty with obesity and related diseases. Factors such as limited access to healthy food, reduced opportunities for physical activity, and chronic stress explain this relationship. It is essential for public policies to promote access to nutritious foods, environments that facilitate exercise, and strategies to overcome structural barriers in low-income communities. Additionally, the marketing of ultra-processed foods in vulnerable areas should be regulated. This comprehensive approach could reduce inequalities and improve health outcomes for disadvantaged populations.

Among the most novel contributions of this study is the observation that smokers had significantly higher levels of visceral fat. This finding supports the hypothesis that smoking contributes to an adverse metabolic profile, increasing the risk of abdominal obesity and associated metabolic diseases. Smoking, in addition to its well-known negative effects on cardiovascular and pulmonary health, seems to exacerbate the accumulation of visceral fat, adding a metabolic component to the harm caused by this habit. Therefore, including smoking cessation programs as an integral part of obesity management strategies could have a significant impact on improving the metabolic health of individuals and, ultimately, the population at large.

Obesity generates high demand for medical care due to disabilities associated with non-communicable diseases such as type 2 diabetes, cardiovascular diseases, and certain cancers, significantly increasing healthcare costs. Addressing modifiable risk factors, such as unhealthy diets, physical inactivity, smoking, and harmful alcohol consumption, is essential to improving public health and reducing costs.

Our study highlights physical inactivity as a key determinant in the development of obesity and visceral fat. Promoting physical activity from early ages and in environments like the workplace, along with encouraging balanced diets like the Mediterranean diet, can prevent these conditions. Early prevention not only improves quality of life but also reduces long-term medical costs.

Preventive interventions, such as educational programs, awareness campaigns, and health promotion policies, should be prioritized. These actions benefit both individuals and society by increasing healthcare efficiency and productivity. In the face of budgetary limitations, it is crucial to analyze the costs associated with obesity to optimize resources and prioritize strategies that promote healthy habits, reduce smoking, and encourage physical activity, contributing to the sustainability of health systems.

### Strengths and Limitations

One of the strengths of this study is its large sample size, which includes nearly 8600 participants, as well as the broad range of variables analyzed.

The primary limitation, however, is that the sample only includes individuals of working age (18–69 years), excluding unemployed individuals, retirees, those under 18, and those over 69 years old. As a result, our findings cannot be generalized to the entire population, as certain age groups are not represented.

Since the sample comes solely from a population in Spain, the results may differ in other populations, so our findings are not generalizable to them.

Other confounding factors, such as comorbidities or pharmacological treatments, were not included, as these data were not available.

## 5. Conclusions

Age, sex, social class, tobacco use, and physical activity are factors that show a high association with body and visceral fat levels. Understanding how these factors are associated with fat distribution is crucial for the development of personalized prevention and treatment strategies that can mitigate the risk of obesity-associated diseases. Given that visceral fat is an independent predictor of metabolic risk, addressing these factors could have a significant impact on public health. Future research should focus on further elucidating the interactions between these factors and developing targeted intervention programs to meet the specific needs of different population groups. This may require structural equation studies to show influence rather than association, as we have done here.

## Figures and Tables

**Table 1:** Characteristics of study participants.

	Men n = 4104	Women n = 4486	
	Mean (SD)	Mean (SD)	*p*-Value
**Age (years)**	41.6 (10.6)	41.5 (10.5)	0.492
**Height (cm)**	175.8 (7.2)	162.5 (6.1)	<0.001
**Weight (kg)**	81.2 (14.8)	63.9 (13.6)	<0.001
**Waist circumference (cm)**	89.8 (12.5)	77.0 (12.0)	<0.001
**Hip circumference (cm)**	101.8 (8.7)	99.6 (10.9)	<0.001
**Systolic blood pressure (mmHg)**	128.6 (13.3)	117.2 (14.1)	<0.001
**Diastolic blood pressure (mmHg)**	79.9 (10.2)	74.9 (9.9)	<0.001
**Glycemia (mg/dL)**	93.4 (17.8)	88.9 (12.6)	<0.001
**Total cholesterol (mg/dL)**	191.8 (36.0)	189.0 (34.8)	<0.001
**HDL-cholesterol (mg/dL)**	49.2 (11.3)	59.5 (12.8)	<0.001
**LDL-cholesterol (mg/dL)**	124.0 (54.6)	113.8 (30.7)	<0.001
**Triglycerides (mg/dL)**	107.8 (69.4)	81.5 (46.3)	<0.001
**GGT (UI)**	31.5 (30.0)	18.5 (15.9)	<0.001
**AST (UI)**	24.4 (17.3)	18.2 (7.7)	<0.001
**ALT (UI)**	29.3 (34.9)	17.3 (13.4)	<0.001
	**%**	**%**	***p*** **-value**
**18–29 years**	15.5	16.8	0.005
**30–39 years**	27.8	25.1	
**40–49 years**	32.7	34.4	
**50–59 years**	19.0	19.7	
**60–69 years**	5.0	4.0	
**Social class I**	57.1	50.8	<0.001
**Social class II**	20.2	23.8	
**Social class III**	22.7	25.4	
**Non-smokers**	84.5	84.2	0.348
**Smokers**	15.5	15.8	
**Low physical activity**	25.9	35.1	<0.001
**Physical activity 1–** **3 days/week**	27.0	26.5	
**Physical activity more 3 days/week**	47.1	38.4	

HDL, high-density lipoprotein. LDL, low-density lipoprotein. GGT, gamma-glutamyl transpeptidase. Social class I (upper class). Social class II (middle class). Social class III (lower class). AST, aspartate aminotransferase. ALT, alanine aminotransferase. SD, standard deviation.

**Table 2:** Mean values of body fat and visceral fat according sociodemographic variables and healthy habits.

		Men					Women			
		Body Fat		Visceral Fat			Body Fat		Visceral Fat	
	n	Mean (SD)	*p*-Value	Mean (SD)	*p*-Value	n	Mean (SD)	*p*-Value	Mean (SD)	*p*-Value
**18–29 years**	636	15.2 (7.0)	<0.001	3.4 (2.9)	<0.001	754	24.9 (6.6)	<0.001	2.1 (2.2)	<0.001
**30–39 years**	1140	18.1 (6.5)		6.0 (3.6)		1126	27.0 (7.3)		3.3 (2.5)	
**40–49 years**	1344	20.4 (8.4)		8.6 (4.0)		1544	30.3 (7.6)		5.2 (2.8)	
**50–59 years**	780	23.8 (6.4)		11.8 (3.9)		882	33.0 (7.4)		7.1 (2.8)	
**60–69 years**	204	27.5 (6.1)		13.2 (3.7)		180	34.2 (6.9)		8.4 (3.3)	
**Social class I**	2346	18.6 (7.2)	<0.001	7.0 (4.6)	<0.001	2278	26.6 (7.1)	<0.001	3.5 (2.6)	<0.001
**Social class II**	828	21.5 (9.0)		8.9 (4.7)		1068	31.9 (7.5)		5.7 (3.1)	
**Social class III**	930	22.1 (7.7)		9.3 (4.8)		1140	32.1 (7.9)		6.2 (3.6)	
**Non-smokers**	3468	19.7 (8.0)	<0.001	7.8 (4.8)	<0.001	3776	29.1 (7.8)	<0.001	4.6 (3.2)	<0.001
**Smokers**	636	21.3 (7.2)		8.0 (4.4)		710	30.1 (8.3)		5.1 (3.3)	
**Low physical activity**	1062	25.2 (8.6)	<0.001	10.7 (5.2)	<0.001	1574	32.7 (7.9)	<0.001	5.9 (3.7)	<0.001
**Physical activity 1–3 days/week**	1110	20.5 (6.2)		8.2 (4.4)		1187	28.4 (7.3)		4.3 (2.9)	
**Physical activity more 3 days/week**	1932	16.8 (6.7)		6.2 (3.9)		1725	26.8 (7.1)		3.9 (2.6)	

SD, standard deviation. Social class I (upper class). Social class II (middle class). Social class III (lower class).

**Table 3:** Prevalence of high values of body fat and visceral fat according to sociodemographic variables and healthy habits.

		**Men**					**Women**			
		**Body Fat Very High**		**Visceral Fat High**			**Body Fat Very High**		**Visceral Fat High**	
	**n**	**%**	***p*-Value**	**%**	***p*-Value**	**%**	***p*-Value**	**%**	***p*-Value**	**%**
**18–29 years**	636	5.7	<0.001	2.8	<0.001	754	2.9	<0.001	0.3	<0.001
**30–39 years**	1140	10.7		6.8		1126	5.9		1.4	
**40–49 years**	1344	13.2		12.5		1544	10.8		2.2	
**50–59 years**	780	22.3		33.8		882	17.0		5.2	
**60–69 years**	204	23.5		61.8		180	20.0		12.2	
**Social class I**	2346	9.7	<0.001	13.0	<0.001	2278	4.2	<0.001	0.9	<0.001
**Social class II**	828	11.6		15.9		1068	15.4		4.5	
**Social class III**	930	24.5		23.2		1140	15.8		4.6	
**Non-smokers**	3468	12.6	<0.001	15.9	<0.001	3776	9.5	<0.001	2.4	<0.001
**Smokers**	636	17.9		16.0		710	11.3		3.9	
**Low physical activity**	1062	31.1	<0.001	32.8	<0.001	1574	18.4	<0.001	5.8	<0.001
**Physical activity 1–3 days/week**	1110	10.8		15.7		1187	6.6		1.9	
**Physical activity more 3 days/week**	1932	5.3		6.8		1725	4.2		0.3	

Social class I (upper class). Social class II (middle class). Social class III (lower class).

**Table 4:** Multivariate logistic regression.

	Body Fat Very High		Visceral Fat High	
	OR (95% CI)	*p*-Value	OR (95% CI)	*p*-Value
**Women**	1		1	
**Men**	1.89 (1.64–2.15)	<0.001	11.76 (9.40–14.13)	<0.001
**18–29 years**	1		1	
**30–39 years**	1.25 (1.20–1.31)	<0.001	3.05 (2.27–3.83)	<0.001
**40–49 years**	2.20 (1.63–2.78)	<0.001	11.29 (8.35–14.23)	<0.001
**50–59 years**	2.30 (1.72–2.88)	<0.001	19.38 (13.84–24.92)	<0.001
**60–69 years**	4.23 (3.33–5.14)	<0.001	45.39 (27.04–63.74)	<0.001
**Social class I**	1		1	
**Social class II**	1.67 (1.39–1.95)	<0.001	1.43 (1.14–1.73)	<0.001
**Social class III**	2.55 (2.15–2.96)	<0.001	1.68 (1.38–2.01)	<0.001
**Non-smokers**	1		1	
**Smokers**	1.19 (1.10–1.29)	<0.001	1.29 (1.20–1.39)	<0.001
**Physical activity more 3 days/week**	1		1	
**Physical activity 1–3 days/week**	3.15 (2.63–3.68)	<0.001	2.97 (2.40–3.45)	<0.001
**Low physical activity**	6.21 (5.17–7.26)	<0.001	7.58 (6.05–9.12)	<0.001

OR, odds ratio. CI, confidence interval. Social class I (upper class). Social class II (middle class). Social class III (lower class).

## Data Availability

The study data are stored in a database that complies with all security measures at the ADEMA-Escuela Universitaria. The Data Protection Delegate is Ángel Arturo López González.
